# Experimental and Numerical Simulation of a Radiant Floor System: The Impact of Different Screed Mortars and Floor Finishings

**DOI:** 10.3390/ma15031015

**Published:** 2022-01-28

**Authors:** Ricardo M. S. F. Almeida, Romeu da Silva Vicente, António Ventura-Gouveia, António Figueiredo, Filipe Rebelo, Eduardo Roque, Victor M. Ferreira

**Affiliations:** 1 Department of Civil Engineering, Polytechnic Institute of Viseu, Campus Politécnico de Repeses, 3504-510 Viseu, Portugal; ralmeida@estgv.ipv.pt (R.M.S.F.A.); ventura@estgv.ipv.pt (A.V.-G.); 2 CONSTRUCT-LFC, Faculty of Engineering (FEUP), University of Porto, Rua Dr. Roberto Frias s/n, 4200-465 Porto, Portugal; 3 RISCO, Department of Civil Engineering, Campus Universitário de Santiago, University of Aveiro, 3810-193 Aveiro, Portugal; ajfigueiredo@ua.pt (A.F.); filiperebelo@ua.pt (F.R.); eroque@ua.pt (E.R.); victorf@ua.pt (V.M.F.); 4 ISISE, Department of Civil Engineering, Campus de Azurém, University of Minho, 4800-058 Guimarães, Portugal

**Keywords:** radiant floor, heat transfer, screed mortar, thermal analysis, finite element method, floor finishing

## Abstract

The radiant floor system market is growing rapidly because Europe is moving toward a low-carbon economy and increased awareness about environmental sustainability and energy efficiency, stimulated by the ambitious EU Energy Efficient Directive and nZEB challenge. The high growth rate of the market share is due to the involvement of homeowners in the specifications of their living commodities, so they are thus willing to invest more at the initial stage to obtain long-term benefits and lower energy exploration costs. We performed an experimental campaign over three slabs with a hydronic radiant floor system of equal dimensions, shape, and pipe pitch with different screed mortar formulations to assess their performance throughout a heating/cooling cycle. The temperature at different heights within the interior of the screed mortars and at the surface were monitored. The results revealed that an improved screed mortar has a relevant impact on the efficiency of the system. Moreover, a three-dimensional transient heat transfer model was validated using the experimental data. The model was used to evaluate the impact of different finishing materials, namely wood, cork, ceramic, and linoleum, on the floor surface temperatures. The results showed differences of 15% in the surface temperature when using different floor finishing solutions.

## 1. Introduction

The ambitious energy efficiency standards driven by European Union (EU) Energy Efficient Directive have impacted the national regulations leading to the challenge of nearly zero energy buildings (nZEB) [1,2,3,4,5]. As indoor heating and cooling are the major sources of buildings’ energy consumption [6], when it comes to indoor heating and cooling, radiant floor systems (RFSs) comprise specific characteristics that play a key role in attaining the EU energy efficiency goals. Considering indoor environment quality, radiant heat transfer improves thermal comfort by preventing cold draughts while mitigating the air-borne noise related to system operation. Additionally, the use of radiant systems result in the reduced transportation of pollutants and allergens compared to convective systems, contributing to healthier indoor environments. In combination with the ability to ensure high thermal comfort, reduced energy consumption, and quiet operation, RFSs became increasingly popular, currently being installed in 30–50% of EU new buildings [7,8]. Additionally, RFSs ensure uniform temperature distribution [9] with low distribution losses [10], and therefore highly efficient heating systems, able to deliver improved thermal comfort with minimal vertical temperature gradient compared to air systems solutions [11].

The RFS market in the EU is experiencing rapid growth as Europe is moving toward a low-carbon economy and an increased awareness about environmental sustainability and energy efficiency. In this sense, radiant heating systems are particularly suitable to be used in combination with heat pumps or solar panels, since RFSs can increase the coefficient of performance of heat pumps while ensuring lower energy consumption and CO_2_ emissions [11,12,13,14]. RFSs are appropriate for both heavyweight and lightweight systems. Both have strengths and weaknesses when indoor heating and cooling is performed through RFSs. Regarding conventional construction, the piping system is usually embedded into a screed layer on a layer of thermal insulation. If indoor temperature requires a fast adjustment, due to the high thermal inertia of wet heavyweight construction, RFSs may experience added difficulties in adequately regulating the indoor temperature in a short period of time [15]. Dry, lightweight construction, on the other hand, due to its reduced thermal inertia, may be able to overcome this limitation, enhancing the performance of the RFS. Zhao et al. [16] studied concrete core radiant floors and light radiant floors in terms of dynamic behaviour under intermittent operation, observing that dry systems present up to six times shorter time of heating and cooling compared to wet systems. Yu et al. [17] developed an experimental study evaluating the steady-state and dynamic performance of lightweight radiant floor panels under heating and cooling conditions, achieving steady-state conditions for dry floor heating in under 30 min. Numerous authors referred to the lack of thermal inertia as a key parameter for achieving thermal comfort in lightweight construction [18,19,20,21].

RFSs have been the subject of numerous studies focusing on the system configuration, material layers, thermal behaviour, numerical simulation methods, and operation strategies. Recent research has been conducted on RFSs and important aspects have been pointed out by the scientific community: system configuration and material layers/components [22,23,24,25,26]; thermal analysis (energy transfer, heating/cooling capacity) and, indirectly, thermal comfort [9,27]; numerical thermal and energy simulation (floor- and building-scale) [28,29,30]; and smarter energy control/operating strategies [31]. Regarding system configuration and material layers, the use of functionalized materials with highly conductive matrices and phase change materials (PCMs) acting as thermal energy storage systems is proving to be a promising strategy toward efficiency and RFS performance. Thus, since RFS perform heating and cooling through large surfaces, incorporating PCM into RFS is stimulating: it increases thermal storage, and the phase change allows the RFS to discharge energy for longer periods [22,32]. Additionally, the PCM latent heat storage properties can contribute to shifting the building’s peak electricity load to off-peak periods [33]. Zhou and He [25] investigated, through experimental research, the performance of a low-temperature radiant floor heating system with different heat storage materials (sand and PCM) and heating pipes. The results indicated the advantages of using a PCM–capillary mat combination for low-temperature floors with hot water heating systems. In this subject an increasing trend was observed in recent years, focusing on thermal conductivity, temperature distribution, heat flux analysis, water system temperatures, setpoint optimization, and energy consumption reduction [9,34,35,36,37,38,39,40]. Concerning the use of innovative materials for enhancing the thermophysical properties of RFS, a current line of research involves the incorporation of industrial by-products into screed mortar [41,42].

With respect to RFS control strategies for improving indoor comfort and energy efficiency, Ren et al. [43] investigated the operational control of radiant floor cooling, considering several factors (including condensation risk [44,45], achieving dynamic optimal control of the radiant cooling system.

The thermal conductivity of the system layers, with special importance concerning the screed mortar layer, plays a significant role in RFS performance since its overall behaviour is highly dependent on the heat transfer within the constituent layers of the system [8]. Depending on the heat exchange between the RFS surface layer (finishing material) and the environment, the heat conduction between the surface and the piping system (screed mortar) and the heat transport by the water [8], the RFS must be able to quickly attain suitable surface temperature distribution to enable thermal comfort. Wu et al. [46] evaluated the thermal conductivity of graphite-based cement concrete. Different graphite contents were used to replace an equivalent mass of fine aggregate. The authors found a thermal conductivity increase of 20% to 50%. Compressive strength was reduced up to 90%. Ding et al. [47] observed that when the layer embedding the piping system in an RFS comprises high thermal conductivity, the heating capacity through the upper floor surface increases, diminishing the influence of pipe spacing on heat flux. Regarding the influence of the thermal conductivity of the finishing material and the pipe spacing on the heating capacity of RFSs, heat flux increases with higher thermal conductivity values and decreases with higher values of pipe spacing. Additionally, the higher thermal conductivity of the finishing material implies a greater influence of pipe spacing on heat flux. Lee et al. [48] analysed the life cycle energy for two different finishing materials, finding that a thin flooring panel resulted in a 7.2% reduction in energy consumption compared to a conventional wooden floor panel for residential buildings. Cholewa et al. [49] studied the effect on the thermal comfort of a room with RFS considering seven finishing material solutions. The results revealed that the use of finishing materials with lower thermal conductivity contributes to a reduction of 40% in the energy efficiency of the RFS.

From the literature, two important observations can be highlighted: the overall efficiency of the RFS can still be improved by enhancing the thermal properties of the screed mortar, and the influence of the finishing materials on the RFS performance is relevant. Thus, the results of an experimental campaign comparing the performance of different screed mortars are herein presented and discussed for reducing the literature gap identified on the topic of screed mortars. Additionally, a sensitivity analysis concerning the impact of different finishing materials in the thermal performance of the RFS was performed using a three-dimensional finite element (FE) model to optimize the performance of an RFS.

## 2. Methodology

This work was developed following a methodology divided into two approaches: (i) experimental work and (ii) numerical simulation. The thermophysical and mechanical properties of the screed mortars were characterized following EN 12664:2001 [50]; ISO 8302:1991 [51] and EN 1015-11 [52]. Once characterized, the experimental work involved two setups focusing on the experimental evaluation of three slabs with a hydronic RFS and different mortar screeds (M_01: innovative formulation; M_02: self-compacting; M_03: traditional screed as reference mortar) with the objective of improving thermal performance and energy efficiency. Setup one concerned the thermal performance, and setup two enabled the evaluation of the energy behaviour of the RFS solutions with the three different mortar screeds. Additionally, the results attained from the experimental campaign were used to calibrate a numerical model developed to perform a sensitivity analysis regarding the effect of different finishing materials applied in a hydronic RFS.

The testing procedures of the two mentioned setups that compose the experimental campaign were as follows:

- Setup 1 (continuous heating): Thermal behaviour analysis of the three slabs when the hot water system was working (turned on) for 5 h and then turned off;
- Setup 2 (intermittent heating): Working period was evaluated by the accumulated hours with the hot water system working (turned on) while subjected to a trigger (on/off) with a setpoint range between 26 and 29 °C.

Regarding the numerical analysis, the reference mortar (M_03: traditional screed mortar) properties were used to assess the impact of applying different finishing layer materials over the screed mortar in terms of the surface temperatures’ distribution. Four common materials were selected to be numerically evaluated: ceramic, linoleum, wood, and cork.

**Figure 1:**
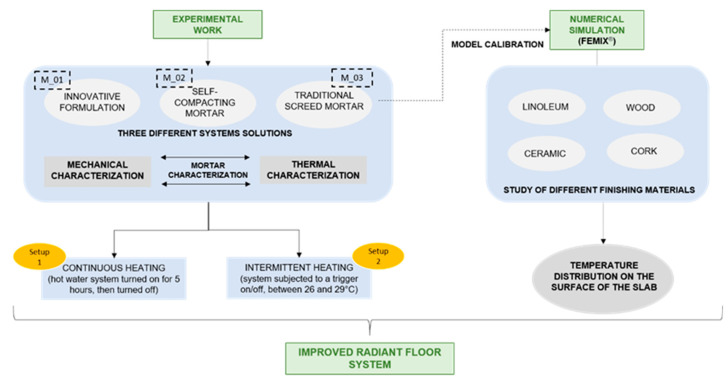
Methodology followed: thermal behaviour improvement of radiant floor systems.

Our workflow for assessing the RFS performance followed the methodology depicted in Figure 1.

## 3. Experimental Setup

An experimental setup was built that included three rectangular slabs with a hydronic RFS of the same size, shape, and pitch. The dimensions of the slabs were 1.20 × 1.50 m, with a thickness of 0.095 m. Hot water piping was applied upon an insulation board and embedded within a screed mortar. Each slab was executed with a different screed mortar formulation (as mentioned in Section 2) and their performance was monitored under heating cycle and free-floating conditions (system turned off).

**Figure 2:**
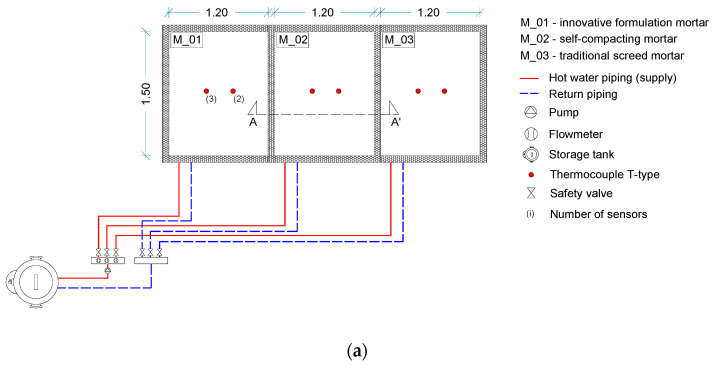

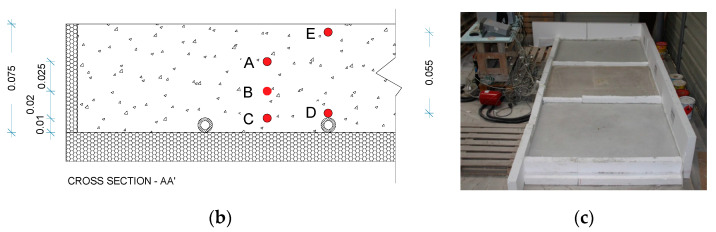
Experimental test setup: (**a**) full layout; (**b**) cross-section with thermocouples’ detailed position; (**c**) full view of the test setup real installation (dimensions in metres).

The temperature at different heights in the interior of the slab (in the screed mortar) and the superficial temperature were continuously monitored during the test using T-type thermocouples. Figure 2 presents the experimental setup, including the details of the positions of the thermocouples.

### 3.1. Materials Characterization

Three screed mortars were used: M_01 was an innovative formulation designed purposefully for higher thermal conductivity; M_02 was a self-compacting mortar; and M_03 was a traditional screed mortar considered as the reference solution. The most relevant properties of the three screed mortars were determined in laboratory conditions: hardened density, thermal conductivity, and mechanical strength for flexural and compressive performance. The values presented in Table 1 are the average taken from four specimen measurements for each property listed. The low standard deviation values corroborate the reliable values attained. 

### 3.2. Setup 1: Continuous Heating

**Figure 3:**
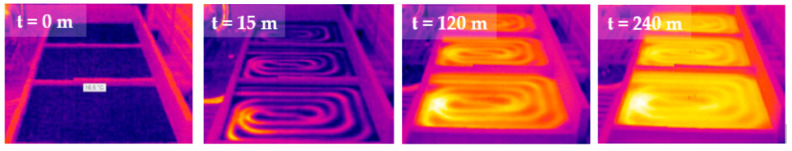
Thermal images taken throughout the heating period (m, minutes).

For setup 1 of the experimental tests, the system was turned on for 5 h and then turned off. During heating, the hot supply water temperature increased from 30 to 45 °C. The surface temperature was also assessed by infrared thermography. Figure 3 presents three examples of thermal images acquired during the heating period.

**Figure 4:**
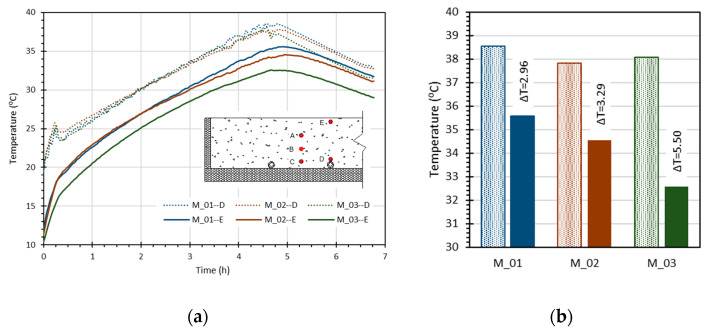
Test results: (**a**) temperature variation and (**b**) maximum temperature reached in the mortars.

As observed in Figure 3, a uniform surface temperature distribution was achieved after 240 min regarding the three test slabs. The temperature distribution within the screed mortar was monitored in five positions (A to E). The temperature values over the pipe (D) and close to the surface (E) are presented in Figure 2b. The temperature variation throughout the test is presented in Figure 4a, and the differences in the maximum temperature attained in each slab are highlighted in Figure 4b.

Figure 4a depicts to the temperature profile evolution of two monitoring points of the screed mortar (D, over the pipe in which water circulates at 45 °C; E, near the slab surface). The temperature profiles highlight that a more rapid temperature rise was recorded for all screed mortar solutions within the first 20 min of heating, indicating a significant energy transfer to the screed mortars that then continued to increase at a slower rate.

During heating, the temperature values of the sensor located in (D) were identical for all three specimens, indicating that the system was working correctly and that the results were comparable. Regarding the effect of thermal conductivity of the screed mortar and analysing the variation in the surface temperature, it was possible to observe the importance of the screed mortar on the heating rate of the mortar layer and the efficiency of the radiant system. As expected, the traditional mortar screed (M_03) revealed lower performance and the mortar screed, with improved thermal conductivity (M_01), managed to reach higher temperatures on the slab surface, thus revealing higher performance. At the end of the heating period, the observed difference in surface temperature between the two slabs was 3.1 °C. Comparing the temperature between the sensor (D) (over the pipe) and the slab surface (sensor (E)), a small difference was observed for the slab with the optimized mortar (M_01), with a value of 2.96 °C, while for the slab with traditional mortar screed (M_03), the observed temperature difference was 5.50 °C.

### 3.3. Setup 2: Intermittent Heating

For setup 2, the test methodology consisted of evaluating energy behaviour as a function of the accumulated working hours with the hot water system working (turned on) for a complete heating and cooling cycle (charge and discharge), taking the RFS with mortar M_03 as the reference.

This setup was of paramount importance since one of the major issues was to fully explore the possibility of significant energy savings without compromising the surface temperatures to ensure internal comfort conditions. Another aspect that is highly linked to the importance of an intermittent heating strategy is the possibility of controlling RFS operation to optimize water supply temperatures and shift working periods to off-peak electricity loads (periods in which the system is turned off).

**Figure 5:**
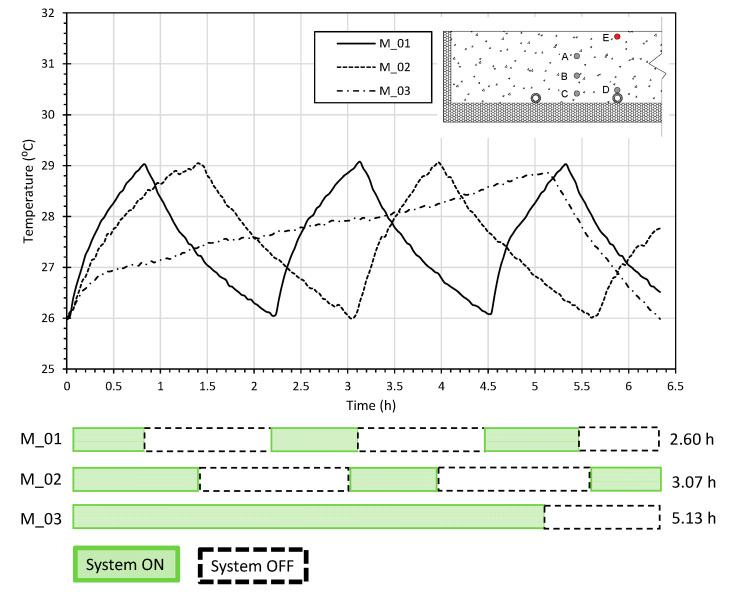
Temperature profile and representative time of system turned on and off for heating (system charge) and discharge (passive behaviour).

The three RFS comprising the different screed mortars under study (M_01, M_02, and M_03) were tested for a temperature setpoint between 26 °C and 29 °C, as shown in Figure 5.

For a complete cycle of the reference mortar (M_03) to be performed, screed mortars M_01 and M_02 performed two complete charge and discharge cycles, requiring reduced working times for the RFS to guarantee an equivalent range of surface temperature (as defined by the setpoint interval of 26 to 29 °C). The RFS with the reference screed mortar M_03 worked for a total accumulated time of 5.13 h. Concerning the RFSs with screed mortar M_01 and M_02, we observed a reduction in the accumulated hours with hot water supply on of 49.3% and 40.2%, corresponding to 2.60 and 3.07 h, respectively. Additionally, a quicker response was observed for M_01 in achieving the maximum setpoint temperature (29 °C), due to the higher thermal conductivity of this specific screed mortar.

Extrapolating the attained results to a monthly basis analysis, the energy consumption expressed by the accumulated hours of the system when turned on experienced an expected reduction of about 284 and 231 h month^−1^ for M_01 and M_02, respectively, both in comparison with the expected operating time of the reference solution for M_03.

## 4. Thermal Simulation

In the numerical analysis, the M_01 slab, executed with the traditional mortar screed, was simulated to evaluate the impact of applying different materials on the slab surface as a finishing layer, by analysing the surface temperatures’ distribution. The numerical simulation of the hydronic RFS was performed using FEMIX computer software [53]. A general thermal model to simulate the heat transfer in structures built with materials whose mechanical behaviour can be considered linear or nonlinear is available in FEMIX, including heat development due to the hydration process of cement-based materials [54]. Thus, this general thermal model is specified for steady-state and transient thermal analysis as well as for nonlinear thermal analysis. This thermal model can be coupled with a mechanical model to simulate specific thermophysical and mechanical aspects such as crack initiation and propagation in structures discretized with solid finite elements [55]. Since the mechanical analysis was beyond of the scope of this research, only the thermal behaviour of the slab system was analysed and compared to the obtained experimental data.

The general three-dimensional heat conduction equation in Cartesian coordinates can be presented as follows:

(1) $$\frac{\partial }{\partial x}\left(\lambda_{x}\frac{\partial T}{\partial x}\right)+\frac{\partial }{\partial y}\left(\lambda_{y}\frac{\partial T}{\partial y}\right)+\frac{\partial }{\partial z}\left(\lambda_{z}\frac{\partial T}{\partial z}\right)+\dot{Q}=\rho_{c}\frac{\partial T}{\partial t}$$

For the case of isotropic materials, the thermal conductivity is the same in all directions, i.e., *λ_x_ = λ_y_ = λ_z_ = λ*. $\dot{Q}$ is the internal heat generation rate per unit volume of the infinitesimal control volume, *ρ* is the mass per unit volume, and *c* is the specific heat of the material.

To obtain the temperature distribution in a body, the heat conduction equation must be solved considering appropriate boundary conditions such as prescribed temperature in the boundary, constant heat flux in the boundary, insulated or adiabatic boundary, and convection and/or radiation condition on the boundary surface. For the case of time-dependent temperature phenomena, the initial conditions must also be known. In the present simulation, prescribed temperatures to simulate the water temperature and convection condition on the top surface of the slab were used.

**Figure 6:**
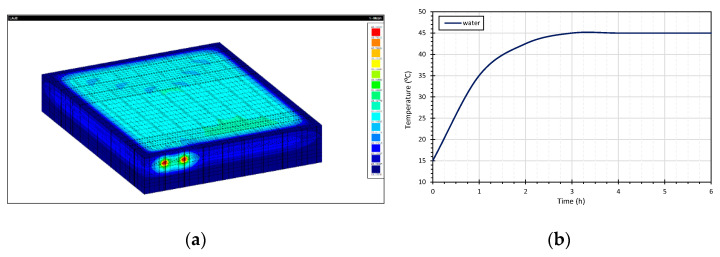
(**a**) FE three-dimensional model; (**b**) water temperature profile.

**Figure 7:**
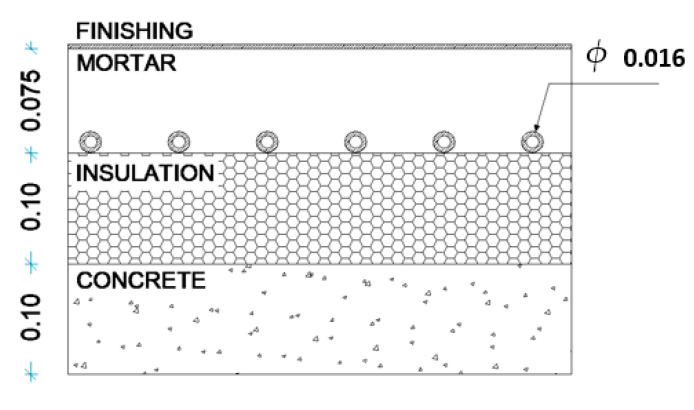
Cross-section of the FE model (dimensions in metres).

Solid hexahedra finite elements (FEs) of 8 nodes were used to simulate the slab. The adopted mesh comprised 31,200 elements and 35,139 nodes (Figure 6a). To simulate the test conditions, the ambient air temperature was defined according to the measurements carried out in situ and the water temperature was imposed as presented in Figure 6b. Figure 7 is a schematic representation of the cross-section of the slabs used in the modelling, and Table 2 presents the material properties introduced in the model.

**Figure 8:**
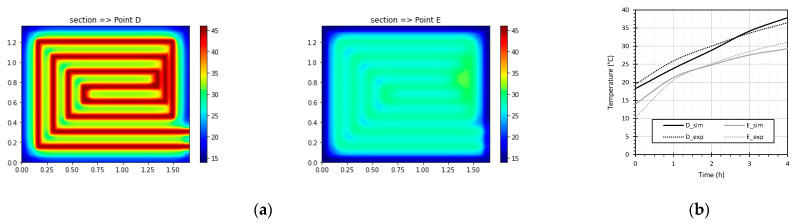
(**a**) Temperature distribution in two sections (point (D) and point (E)); (**b**) comparison between recorded and simulated data.

The FE model was initially validated. To this end, the simulated temperature values over the tube and near the surface were compared to the results recorded during the test at monitoring points (D) and (E). Figure 8a presents the results of the temperature distribution at the end of the test in the sections corresponding to monitoring points positioned in (D) and (E), and Figure 8b compares the measured and simulated temperature values. The slight differences between the simulation results and the measured temperature values can partly be explained by the unavoidable uncertainty associated with the positioning of the thermocouples and, consequently, with the choice of the closest node in the FE model. In future work, the model will be further improved through an increase in the level of discretization of the mesh, especially in the areas surrounding the tube where the highest temperature gradients occur. Nevertheless, the accuracy found in the FE model was compatible with the purpose of our research.

**Figure 9:**
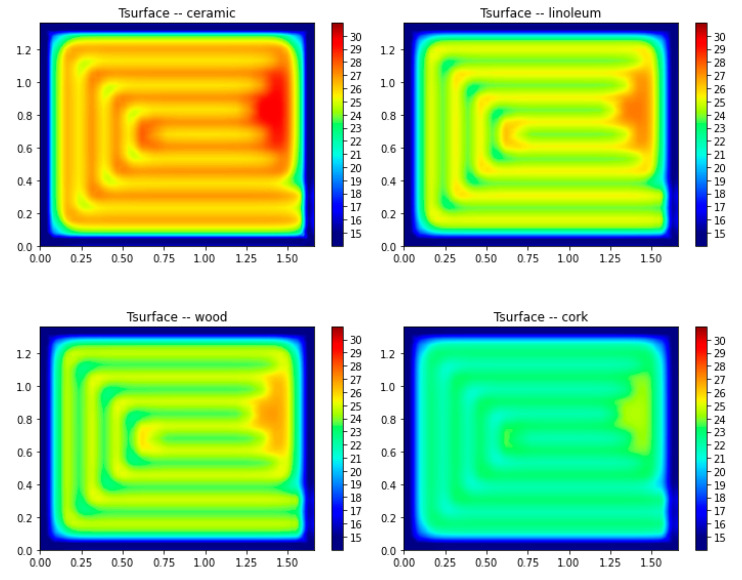
Temperature distribution in the surface of the slab considering different finishing materials (results in degrees Celsius).

After validation, the model was used for a sensitivity analysis to assess the impact of using different finishing materials in the distribution of surface temperatures. As such, four typical materials applied in RFS were selected: ceramic, linoleum, wood, and cork. The properties of the materials used in the simulation are detailed in Table 3 as well as the obtained temperatures regarding the maximum (T_max_) and the average temperature of the surface (T_surf_). Figure 9 shows the temperature distribution on the surface of the slab for the different finishing materials.

The results confirmed the importance of the finishing material in terms of the temperature reached on the surface and, consequently, the efficiency of radiant floor heating systems. The maximum temperature reached on the surface varied between 24.56 and 29.76 °C. The thermal properties of the coating material led to differences of 15% in the average surface temperature of the slab.

**Figure 10:**
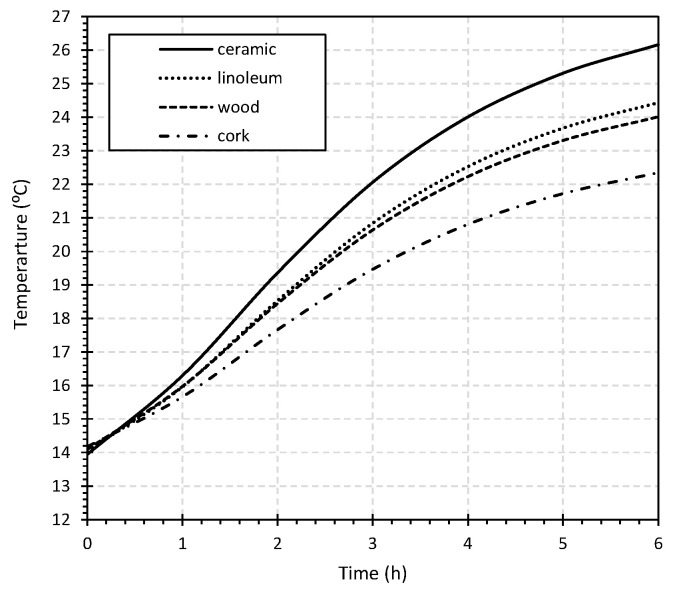
Surface temperature evolution profile for the different finishing materials.

A central point of the FE mesh was selected for a detailed analysis of the surface temperature evolution profile considering the application of the different finishing materials, as presented in Figure 10.

As observed in Figure 10, the use of ceramic as the finishing material of an RFS provides the advantage of higher thermal conductivity, resulting in higher temperatures and reduced operation time. Ceramic finishing reached the same temperature value approximately two hours before the cork finishing. Additionally, after 6 h of operation, the temperature difference between these two finishing materials was 4 °C. Due to the lower thermal conductivity, cork resulted in the finishing material requiring a longer operating period of the system with a lower surface temperature achieved. Linoleum and wood produced similar results in terms of surface temperatures profile.

## 5. Conclusions

This paper presented the results of an experimental campaign and a calibrated numerical model to assess the impact of different floor slab finishing materials.

From the results of the first setup, the importance of the thermal properties of the screed mortar on the heating and cooling rate of the RFS was evidenced. In the conditions under which the test was performed, in terms of thermal conductivity, a difference of more than 3 °C in the maximum temperature was reached for all three screed mortar used in the slabs.

Concerning the intermittent heating setup, the better performance of the screed mortar M_01 led to the fewest working hours when subjected to setpoint range between 26 and 29 °C, corresponding to a reduction of 49.3% in comparison to the reference mortar (M_03). Concerning the screed mortar M_02, a reduction of 40.2% was observed compared to M_03. This enhanced performance, extrapolated to a monthly analysis, can lead to a significant reduction in operating time for M_01 and M_02 in comparison to M_03.

Once the numerical model was calibrated, a sensitivity analysis was carried out referencing o the thermal simulation of the performance of the slabs with a three-dimensional FE model to assess the importance of the finishing material in the overall efficiency of the RFS. From the simulation results, the surface temperature difference between the cork and ceramic finishing materials after 6 h of operation was approximately 4 °C. Considering a surface temperature value of 22 °C, the use of a ceramic finishing allows reaching this surface temperature approximately two hours earlier than when cork finishing is used.

The chosen thermal conductivity of the screed mortar and the finishing material to be applied in RFS were found to play a significant role in the overall performance of these systems, strongly affecting surface temperatures and operating hours, thus affecting overall energy consumption. Moreover, to achieve the high-end performance of an RFS, other variables in addition to the ones explored herein should also be assessed in future work, such as water supply temperature and flow rate, and the energy storage capability that could be given to some of the system layers with the use of phase change materials.

## Figures and Tables

**Table 1:** Thermal and mechanical properties of the screed mortars: average values (standard deviation).

Mortar	Density (kg m^−3^)	Thermal Conductivity (Wm^−1^ °C^−1^)	Strength (MPa)	
			Flexural	Compressive
M_01	2130 (20.01)	0.817 (0.077)	4.42 (0.15)	16.09 (1.63)
M_02	2110 (16.40)	0.805 (0.073)	6.05 (0.25)	27.53 (1.22)
M_03	2170 (17.10)	0.537 (0.043)	5.54 (0.33)	22.77 (0.36)

**Table 2:** Properties of the materials in the FE model.

Material	Density (kg m^−3^)	Thermal Conductivity (Wm^−1^ °C^−1^)	Specific Heat (J kg^−1^ °C^−1^)
Mortar	2170	0.537	800
Insulation	70.5	0.037	1000
Concrete	2500	2.0	1000

**Table 3:** Properties of the finishing materials and simulation results.

Material	Density (kg m^−3^)	Thermal Conductivity (Wm^−1^ °C^−1^)	Specific Heat (J kg^−1^ °C^−1^)	T_max_ (°C)	T_surf_ (°C)
Ceramic	2300	1.300	840	29.76	26.11
Linoleum	1390	0.170	900	27.54	24.75
Wood	500	0.130	1600	26.88	24.32
Cork	400	0.065	1500	24.56	22.60

## Data Availability

The data presented in this study are available on request from the corresponding author. The data are not publicly available since this research work is still ongoing, based on the attained results.
